# Natural Disasters Are Prejudiced Against Disadvantaged and Vulnerable Populations: The Lack of Publicly Available Health‐Related Data Hinders Research at the Cusp of the Global Climate Crisis

**DOI:** 10.1029/2019GH000219

**Published:** 2020-01-14

**Authors:** Omid Mazdiyasni, Amir AghaKouchak

**Affiliations:** ^1^ Department of Civil and Environmental Engineering University of California Irvine CA USA; ^2^ Department of Earth System Science University of California Irvine CA USA

**Keywords:** climate change, health, natural disasters, climate impacts, social impacts

## Abstract

Natural disasters often affect the most vulnerable countries/communities around the world. However, within the same countries/communities, the impact of natural disasters is far greater on disadvantaged populations. We investigate how wildfires affect asthma prevalence in different populations across California. Our results indicate that although there is no discernible relationship between wildfires and asthma prevalence for California's population as a whole, wildfires and asthma prevalence in Black and senior populations have a strong relationship. We believe there is an urgent need to make high‐resolution health‐related data publicly available for in‐depth analyses of climate change impacts on society and disadvantage communities

## Key Points

- The impact of natural disasters is greater on disadvantaged and vulnerable populations with respect to the overall population
- There is a need to make high‐resolution health‐related data publicly available for in‐depth analyses of climate change impacts on society

## 1 Introduction

Natural disasters often have significant social and economic consequences on populations all over the globe. However, individual communities are impacted in different ways, where poorer and more vulnerable people often experience the worst disasters (Fritz, 1961; Iyengar & Hahn, 2007; West & Orr, 2007). In fact, scientists have noted that disasters discriminate when distributing risk and vulnerability to preexisting social systems (Couch & Kroll‐Smith, 1985; Fordham, 2008). Disasters are often a function of social, political, and economic conditions, in conjunction with the natural events that cause them (Fothergill et al., 1999; Fothergill & Peek, 2004; Spence et al., 2007).

Communities with greater risk factors (e.g., old age) are often more vulnerable to additional hazards. For example, a study found that neighborhoods in New York City with higher asthma hospitalization rates had 5 times more public housing units (housing designed for low‐income residents), when compared to neighborhoods without elevated asthma rates (Corburn et al., 2006; Northridge et al., 2010). However, studies have not investigated how natural disasters affect the difference in likelihood of health hazards for different populations.

In this study, we strive to investigate how natural disasters affect health hazards of different populations across California. We analyze the impacts of wildfires on asthma prevalence on different subgroups, to determine if there is any difference between the impacts of wildfires on underrepresented and vulnerable populations. Since asthma is often triggered by environmental factors, such as air pollution and allergens, smoke and particles released due to wildfires will theoretically have an effect on asthma prevalence (Anenberg et al., 2017; Gan et al., 2017; Horwell & Baxter, 2006).

**Figure 1:**
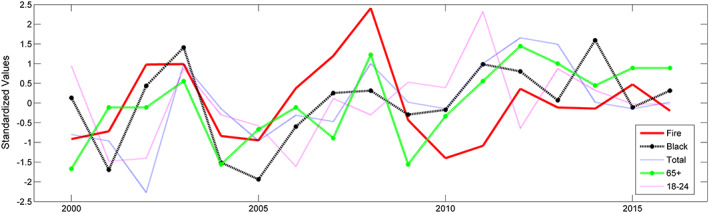
Standardized time series for fire and asthma prevalence for individual subgroups (total population, Black population, senior population, and young adult population).

When comparing annual asthma prevalence with annual wildfires (total burned area for fires >200 ha), we notice there is virtually no relationship. In fact, there is no correlation between California wildfires and asthma prevalence for the entire population across California. However, when analyzing the impact of wildfires on Black populations, we can see that there is an increase in the correlation to nearly 0.4. Figure 1 shows the standardized time series of annual burned area and asthma rates for the total population and Black population, and it is clear that Black populations see a spike in asthma prevalence during years with large burned area due to wildfires.

We also compared the impacts of wildfires on young adult (18–24 years old) and senior (65+ years old) populations. Similar to the overall population, we noticed that young adult asthma prevalence was not affected by wildfires. However, senior populations had a statistically significant correlation of 0.43 with wildfire burned area. Similar to Black populations, we see a peak in asthma rates during significant wildfire years, such as 2003 and 2008.

**Figure 2:**
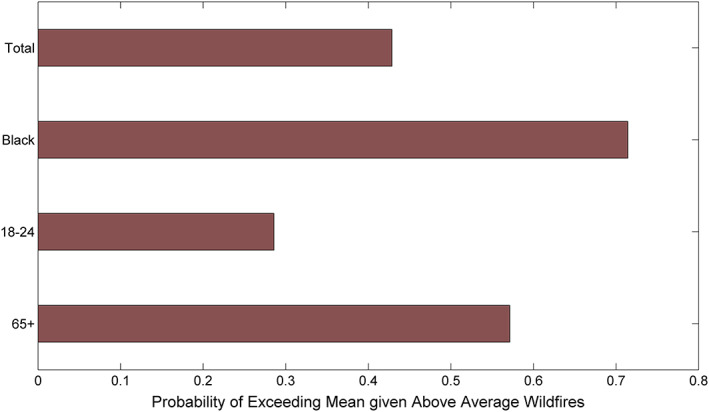
Probability of above average asthma prevalence rates, given above average wildfire burn area across California.

To portray the impacts of wildfires on asthma prevalence, we analyzed the likelihood that different subgroups would experience asthma given certain wildfire conditions. Figure 2 depicts the probability that the total, Black, senior, and young adult populations would have above average asthma prevalence, conditioned on above average wildfire burned area. This figure shows a significant divergence between Black populations and total populations, with the Black population having an approximately 70% higher likelihood in the probability of above average asthma rates given above average wildfires (42% vs. 71%). This figure also shows that senior populations have twice the probability of having above average asthma rates during above average wildfire years (28% vs. 57%).

Studies on natural disasters often analyze global trends and impacts in different regions across the globe. However, there is a gap in studying the impact of natural hazards on various communities in the same geographic location. The impact of natural disasters on individual demographic groups must be analyzed to protect more vulnerable populations in the face of climate change.

For example, California has Black population of approximately 2.3 million based on the 2010 U.S. Census Bureau, and the increasing severity and frequency of wildfires will result in more prevalent asthma rates in this demographic subgroup. Furthermore, advances in technology and health sciences will lead to higher life expectancy, resulting in an increase in senior populations. A higher number of senior individuals will be exposed to wildfire smoke, increasing the asthma rates in these vulnerable populations. We believe that the effects of climate change on demographic subgroups have been overlooked and call on scientists to focus their research on how the changing dynamics of natural disasters will influence not only different regions but also different communities of the same region.

One of the main factors preventing health‐related hazard impact assessment is the availability of hospitalization and health‐related data. Although spatially aggregated climate and hazard data are abundant, health‐related data are scarce and limited in spatial and temporal resolution. It is understandably imperative to keep patient data confidential; however, there are methods to disclose the information necessary for data analysis through data transformation and standardization. As our understanding of the changes in extreme events and natural disasters due to climate change increase, it is important to model the related impacts on human health. For example, Mazdiyasni et al. show that the probability of mass heat‐related mortality rises by approximately 150% with 0.5 °C increase in summer mean temperature(Mazdiyasni et al., 2017); however, due to the limited resolution in the heat‐related mortality data, this analysis may be highly uncertain. Similarly, this study investigated wildfires and asthma across the entire state of California; however, stronger relationships may be present if spatially aggregated data were available. The lack of (or limited) publicly available health‐related data hinders research on natural hazards and climate change impacts on public health and disadvantage communities. Publicly available, high‐resolution, but personally unidentifiable, health‐related data are crucial for rigorous analysis of the impacts of climate change on society, and there must be a push to make these data publicly available.

## 2 Methods

We utilize the correlation coefficient to describe the relationship between wildfires and asthma prevalence for different populations in California. We use a significance level of 0.1 to determine statistically significant correlations.

### 2.1 Standardization

In Figure 1, we standardize all our variables (fire and asthma rates) for better visualization. Standardization transforms the data to have a mean of 0 and a standard deviation of 1. The formula for standardization is written as

$$\text{Standard Variable}=\frac{\text{variable}-\text{mean}\left(\text{variable}\right)}{\text{stdev}\left(\text{variable}\right)}$$

### 2.2 Conditional Probability

In this paper we use a conditional probability approach to determine the probability of above average asthma rates, given above average wildfire burn area. We define the conditional probability as

$$\mathrm{Pr}\left(\text{asthma}>\text{mean}\left(\text{asthma}\right)|\text{fire}>\text{mean}\left(\text{fire}\right)\right)=$$

$$\frac{\mathrm{Pr}\left(\text{asthma}>\text{mean}\left(\text{asthma}\right)\cap \text{fire}>\text{mean}\left(\text{fire}\right)\right)}{\mathrm{Pr}\left(\text{fire}>\text{mean}\left(\text{fire}\right)\right)}$$

where *asthma* is the annual asthma prevalence, *mean* (*asthma*) is the average asthma threshold, *fire* is the annual large fire burn area, and *mean* (*fire*) is the average burn area threshold for *fire*.

### 2.3 Data

We obtained asthma prevalence data from the Centers for Disease Control and Prevention (CDC) Asthma Surveillance data. The asthma prevalence data we obtained has an annual temporal resolution and ranges from the year 2000–2016. The data were collected by the CDC through the Behavioral Risk Factor Surveillance System, the world's largest telephone survey. For each year of the Behavioral Risk Factor Surveillance System asthma data, the CDC asked two questions: 1) Has the person ever been diagnosed by asthma? and 2) Does the person currently have asthma? In this paper, we use the data from the second question to determine impacts of wildfire on sufferers of asthma. More information on asthma can be found online (at: https://www.cdc.gov/asthma/brfss/default.htm).

The data show that the difference in asthma prevalence across each subgroups is minimal: overall population (7.8%), Black population (10.8%), young adult (8.4%), and senior (8.1%).

We analyzed large fires burned area (>300 acres) using data from the CalFire website because we believe burned area from large fires will have the highest relationship with smoke and particles that may affect asthma rates across the state. The data show annual total acres burned for large fires across California from the years 2000–2016. In compliance with AGU's data access policy, we have provided the large fires burned area in Table S1 of the supporting information.

## Conflict of Interest

The authors declare no conflicts of interest relevant to this study.

## Supporting information

Supporting Information S1
