# Supplementary material for: Natural Disasters Are Prejudiced Against Disadvantaged and Vulnerable Populations: The Lack of Publicly Available Health‐Related Data Hinders Research at the Cusp of the Global Climate Crisis
Source: Geohealth. 2020 Jan 14;4(1):e2019GH000219. doi: 10.1029/2019GH000219 (PMC8456234; doi:10.1029/2019GH000219)
Supplement: Supplementary file 1 — Supporting Information S1 [file GH2-4-e2019GH000219-s001.docx]

| Year | Total Acres Burned |
| --- | --- |
| 2000 | 234708 |
| 2001 | 311075 |
| 2002 | 969890 |
| 2003 | 974169 |
| 2004 | 264988 |
| 2005 | 222538 |
| 2006 | 736022 |
| 2007 | 1054655 |
| 2008 | 1525074 |
| 2009 | 422147 |
| 2010 | 46824 |
| 2011 | 168545 |
| 2012 | 730733 |
| 2013 | 546298 |
| 2014 | 535318 |
| 2015 | 773415 |
| 2016 | 514067 |

Table S1: This table shows large fires burned area (> 300 acres) area in acres across California from 2000 to 2016. This data was obtained from the CalFire website.
